# Cryptococcal antigenemia and its predictors among HIV infected patients in resource limited settings: a systematic review

**DOI:** 10.1186/s12879-020-05129-w

**Published:** 2020-06-11

**Authors:** Awoke Derbie, Daniel Mekonnen, Yimtubezinash Woldeamanuel, Tamrat Abebe

**Affiliations:** 1 Department of Medical Microbiology, College of Medicine and Health Sciences, Bahir Dar University, Bahir Dar, Ethiopia; 2 Centre for Innovative Drug Development and Therapeutic Trials for Africa (CDT-Africa), Addis Ababa University, Addis Ababa, Ethiopia; 3 Department of Health Biotechnology, Biotechnology Research Institute, Bahir Dar University, Bahir Dar, Ethiopia; 4 Department of Medical Microbiology, Immunology and Parasitology, School of Medicine, College of Health Sciences, Addis Ababa University, Addis Ababa, Ethiopia

**Keywords:** Cryptococcal antigenemia, Predictors, Resource limited settings

## Abstract

**Background:**

Cryptococcosis is an opportunistic fungal infection that primarily affects people with advanced HIV/AIDS and is an important cause of morbidity and mortality around the globe. By far the most common presentation of the disease is cryptococcal meningitis (CM), which leads to an estimated 15–20% of all HIV related deaths worldwide, 75% of which are in sub-Saharan Africa. However, to the best of our knowledge there is quite limited reviewed data on the epidemiology of cryptococcal antigenemia in a large HIV-infected population in resource limited settings.

**Methods:**

Articles published in English irrespective of the time of publication were systematically searched using comprehensive search strings from PubMed/Medline and SCOPUS. In addition, Google Scholar and Google databases were searched manually for grey literature. Two reviewers independently assessed study eligibility, extracted data, and assessed risk of bias. The pooled prevalence of cryptococcal antigenemia was determined with 95% confidence interval (CI).

**Results:**

**A**mong 2941 potential citations, we have included 22 studies with a total of 8338 HIV positive individuals. The studies were reported in ten different countries during the year (2007–2018). Most of the articles reported the mean CD4 count of the participants below 100 cells/μl. The pooled prevalence of cryptococcal antigenemia at different CD4 count and ART status was at 8% (95%CI: 6–10%) (ranged between 1.7 and 33%). Body mass index (BMI) < 18.5 kg/m^2^, CD4 count < 100 cells, patients presenting with headache and male gender were reported by two or more articles as an important predictors of cryptococcal antigenemia.

**Conclusions:**

Implementing a targeted screening of HIV patients with low BMI, CD4 count < 100 cells, having headache and males; and treatment for asymptomatic cryptococcal disease should be considered. Additional data is needed to better define the epidemiology of cryptococcal antigenemia and its predictors in resource limited settings in order to optimize the prevention, diagnosis, and treatment strategies.

## Background

According to the 2019 United Nations Programme on AIDS (UNAIDS) report, around 37.9 million people globally were living with HIV in 2018. In the same year, about 1.7 million people were newly infected and 700, 000 people died from AIDS-related illnesses globally [1]. Cryptococcosis is one of the most important opportunistic infections among people living with advanced AIDS having defective cellular immune component and is a major contributor to AIDS-related mortality worldwide [2]. In spite of the increasing availability of antiretroviral treatment (ART), cryptococcal disease continues to be a leading cause of death among HIV infected patients in the developing world [3–5]. Considerable number of HIV-infected population still presents late to care with advanced AIDS [6, 7].

The burden of the disease is greatest in middle and low-income countries where there is a high prevalence HIV infection [8–13]. Patients taking immunosuppressive drugs and some immunocompetent hosts are also at risk [14]. Although the infection begins in the lungs, certainly the most common presentation of cryptococcal disease is cryptococcal meningitis (C M) which accounts for 15–20% of all AIDS-related deaths globally, three quarter of which are in sub-Saharan Africa. Coupled with loose adherence to ART and retention in HIV care, an estimated 223, 100 cases of CM resulted in 181, 100 deaths among people living with HIV in 2014 [2, 4, 7, 13, 15]. Screening patients for subclinical cryptococcal infection at the time of entry into ART programs using point-of-care tools like, cryptococcal antigen (CrAg) immunoassays is highly effective in identifying patients at risk of developing CM, allowing these patients to then be targeted with pre-emptive antifungal therapy to prevent the development of severe disease and mortality [16].

Cryptococcal infection is primarily caused by *Cryptococcus neoformans* and *C. gattii* species [8, 9]. *C. neoformans* is encapsulated yeast that can be found in pigeon droppings which causes mild to severe infections like meningitis or disseminated disease in individuals with impaired immunity [10]. The yeast demonstrates several well-characterized virulence factors that contribute to the success of infection. To mention the common one; tolerance to mammalian body temperature at 37 °C, owning a polysaccharide capsule that protects the yeast from phagocytosis, and a thick cell wall with the deposition of phenolic melanin, which has been proposed to protect the yeast from oxidation [11, 12].

While the gold standard for diagnosis of cryptococcal disease is culture from bodily fluids, CrAg test is used to presumptively diagnose the disease with sensitivity and specificity close to 100%. There are several methods to detect cryptococcal antigen in CSF or plasma/serum: latex agglutination (LA), enzyme immunoassay (EIA), and lateral flow assay (LFA) [6]. Recent advances in point-of-care testing, like CrAg test, has made screening and diagnosis of CM rapid, practical, and affordable and have been improving long-term survival. Targeted screening and pre-emptive treatment programs for CrAg are a cost effective method for reducing early mortality [17].

Some of the independent predictors of positive serum cryptococcal antigenemia includes; CD4(+) T cell counts of ≤100 cells/mm, low body mass index, presenting with neck pain, signs of meningeal irritation, and a recent diagnosis of HIV infection [18–21]. Routine screening of such category of patients may detect cryptococcosis, and hence provide an opportunity for early intervention.

Despite the high burden of cryptococcal meningitis related morbidity and mortality in resource limited settings, reviewed data on prevalence of cryptococcal antigenemia and its predictors is missing [22, 23]. Hence, data is required on this field to inform policy makers for input to tailor intervention measures. Therefore, this systematic review was conducted to describe the the level of cryptococcal antigenemia and its predictors in resource limited settings.

## Methods

### Protocol registration

In accordance with the PRISMA guidelines, this systematic review protocol was registered by the International Prospective Register of Systematic Reviews (PROSPERO) on 01 Feb 2019 with a registration number ‘CRD42019119970’.

### Eligibly criteria

Studies were selected according to the following criteria; *Study design*: observational quantitative studies, like cross-sectional and cohort studies that reported the prevalence of cryptococcal antigenemia and its predictors. *Participants*: We included studies that employed HIV infected people irrespective of gender and the age group who were tested for cryptococcal antigenemia. *Interventions*: our interests were 1) the level of cryptococcal antigenemia, which was defined as the presence of cryptococcal Ag (CrAg) in the blood (serum or plasma) and 2) its predictors, which are to mean factors that are statistically associated with the positive cryptococcal Ag test in the blood. *Setting*: we included studies with the outcome of interest reported in resource-limited settings (countries, listed as low and middle income economic status based on the 2018/19 World Bank report) [24]. *Language and publication*: We considered peer-reviewed journal articles, governmental documents and unpublished articles (thesis) reported in English language irrespective of the year of publication.

### Information sources and search strategy

This review was done following the Preferred Reporting Items for Systematic Reviews and Meta-Analysis Protocols (PRISMA) Guidelines [25]. Research papers were systematically searched in PubMed/Medline and SCOPUS, the last search was conducted on 22th of Dec, 2018. Manual search from Google scholar and Google databases was also done for grey literature. The search terms were developed in line with the Medical Subject Headings (MeSH) thesaurus using a combination of the big ideas (or ‘key terms’) which derived from the research question. The reference lists of retrieved articles were probed (forward and back ward searching) to identify articles that were not retrieved from databases and our manual search. The first two authors; AD and DM searched the articles independently.

The domains of the search terms were *Cryptococcus*, cryptococcal antigenemia, cryptococcal meningitis, cryptococcosis*,* cryptococcal antigen/CrAg, associated factors/risk factors (or predictors), HIV, AIDS and resource-limited setting/countries. We combined cryptococcal antigenemia, cryptococcal meningitis*,* cryptococcosis with the Boolean operator “OR”, and the result was combined with the other terms with “AND”. Full search strategy for the two databases is presented in Supplement 1.

### Study selection

Research papers that reported the level of cryptococcal antigenemia and its epidemiological predictors in the stated settings were included. Searched articles were directly imported and handled using EndNote X5 citation manager (Thomson Reuters, New York, USA). Based on the PRISMA protocol, duplicated articles were excluded and the titles and abstracts of the remaining papers were screened independently for inclusion in full text evaluation by the first two authors. Differences between the reviewers were resolved through discussion. In case of disagreements the decision was determined by the last author.

### Data collection process and data items

Data such as the name of the first author, data collection period and year of publication, country where the study was conducted, mean/median age of the study participants, proportion of male participants, type of the study design, the total number of the study participants, the type of specimen (serum/plasma) used for the CrAg test, the proportion of cryptococcal Ag test result, reported statistically significant predictors for CrAg positive test were extracted from the included articles.

### Quality appraisal

To assess the risk of bias, the two authors independently used the nine items (each score one point) based on the Joanna Briggs Institute (JBI) Critical Appraisal tools [26] for prevalence studies. We assumed that papers that scored > 50% (i.e > 5 of 9 scores) of the weighted value of the tool considered as good quality.

### Data synthesis

The data extracted from the included studies were fed into a Microsoft Excel and were presented in terms of 1) the proportion of cryptococcal antigenemia from each study; 2) meta-analysis was done using STATA 14.0 to determine the pooled prevalence of cryptococcal antigenemia. A systematic narrative synthesis was provided in which summary results were presented using text, table and figures. Descriptive statistics, such as: simple counts, ranges and percentages were used to describe the synthesized data.

## Results

### Search results

From the systematically searched databases and other sources, a total of 2941 articles were retrieved and sequentially screened. After removing the duplicate, 2930 were screened by title then 2865 were removed. Consequently, 36 were removed by abstract and 7 by full text with justifiable reasons. Lastly, a total of 22 studies met our inclusion criteria and included in this review for analysis.

**Fig. 1:**
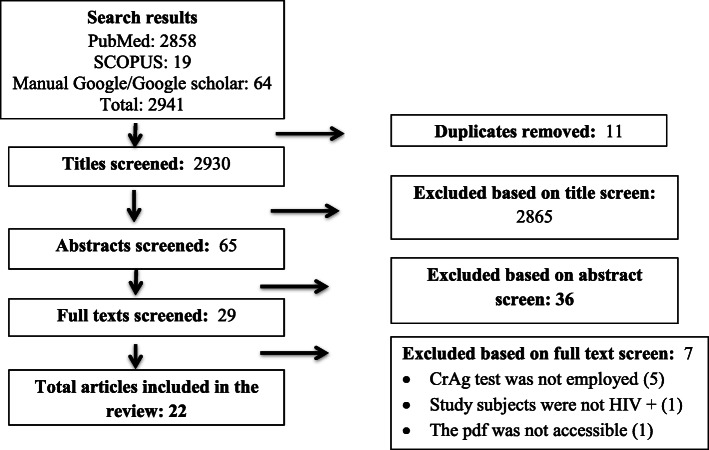
The PRISMA flow diagram of literature selection

Screening was based on the PRISMA flow chart which was adapted from the PRISMA guidelines [27] (Fig. 1).

### Study characteristics

The description of each study is presented in (Table 1). The studies were reported in the last decade (2007–2018) in ten different countries. Except three studies that reported in Brazil, Indonesia and Cambodia, [28, 29, 45] the rest were conducted in Africa. All the included articles were published in peer-reviewed journals. About 19 (86.4%) of the articles used cross-sectional study design; while the remaining three papers were cohort type. The primary interest of most the included papers were to determine the prevalence of cryptococcal antigenemia among HIV infected patients using rapid CrAg test kits.

**Table 1:** Characteristics of the included studies

*Author (s)*	*Pub. Year	*Country*	*Study period*	*Study design*	*Sample size*	*Gender, male (%)*	*Median/* *Mean/ age*
Vidal et al. [28]	2016	Brazil	2014–15	*CS	163	61	38.3
Ganiem et al. [29]	2014	Indonesia	2014	CS	810	76.3	30
Cheryl et al. [30]	2007	Uganda	2003–7	CS	377	29.4	38
Beyene et al. [31]	2013	Ethiopia	2011–12	CS	254	45.3	33
Meya et al. [32]	2010	Uganda	2004–6	CS	609	31	no data
Rugemalila et al. [33]	2013	Tanzania	2011–12	CS	218	43	39
Longley et al. [34]	2016	S. Africa	2011–14	Cohort	645	47	36
Hailu et al. [35]	2017	Ethiopia	2016–7	CS	267	49	38
Letang et al. [36]	2015	Tanzania	2008–12	Cohort	750	40	38
Christopher et al. [37]	2015	Nigeria	2010–11	CS	333	46.8	33
Williams et al. [38]	2015	Uganda	2013–14	CS	207	60.3	36
Alemu et al. [39]	2013	Ethiopia	2011	CS	369	44	36
Derbie et al. [40]	2018	Ethiopia	2016	CS	137	45.3	32
Mamuye et al. [41]	2016	Ethiopia	2013–14	CS	198	53	36.7
Oyella et al. [42]	2012	Uganda	2009–10	CS	367	48	32
Ogouyemi et al. [18]	2016	Benin	2015	CS	355	42.3	40
Drain et al. [43]	2015	S. Africa	2011–13	CS	432	60	36.1
Mdodo et al. [44]	2010	Kenya	2008–9	CS	340	47.5	35
Micol et al. [45]	2007	Cambodia	2004	CS	327	55	35
Jarvis et al. [46]	2009	South Africa	2002–5	CS	707	25	33.5
Wajanga et al. [47]	2011	Tanzania	2009–10	Cohort	333	46.2	38.5
Magambo et al. [48]	2014	Tanzania	2012–13	CS	140	42.1	36

*Pub.year: Publication year *CS: Cross-sectional study design

In this review, data of 8338 HIV positive individuals (male gender 25–76.3% and median age range 30–40 years) were included.

### Risk of bias

The nine domain-based JBI Critical appraisal tool [26] for prevalence studies was used to test outcome level risk of bias of each studies. Each domain had a score of 1 point. The risk of bias for each individual domain was measured as ‘yes’, ‘no’, ‘unclear’ and ‘not applicable’. In this study, ‘yes’ scored 1 and ‘no’ ‘unclear’ and ‘not applicable’ scores zero. The score therefore ranges from zero to nine, with higher scores indicating higher quality of outcome. Based on our assumption the overall score ranged 5–7 (i.e all the included articles scored above 50% positive score). Hence, we considered all as good quality articles.

### Prevalence of cryptococcal antigenemia

The reported median CD4 count was between 20 and 123cell/. Except a study [39] that reported mean CD4 count at 123 cells/μl, the rest reported the mean CD4 count < 100 cells. With regard to ART status of the participants, twelve studies [18, 29, 30, 32, 34, 36, 42, 43, 45–48] included patients who were ART naïve. In contrast, two studies [37, 40] included participants who were on ART. The remaining articles reported different proportion of ART status of the participants. In addition, the reported proportion of headache (ranged between 10 and 80.6%) and WHO clinical stage IV AIDS (ranged between 17.9 and 100%) by the included articles is depicted in Table 2.

**Table 2:** The prevalence of cryptococcal antigemia and distribution of other clinical features of the study participants, 2007–2018

*Author (s)*	*Median CD4 count* (cells/μl)	*ART status*	*WHO stage IV* (%)	*Had headache (%)*	+ *CrAg* test*, n (%)*
Vidal et al. [28]	25	74% on ART	66	No data	5 (3.1)
Ganiem et al. [29]	20	All naïve	no data	No data	58 (7.1)
Cheryl et al. [30]	50	All naïve	36.2	No data	22 (5.8)
Beyene et al. [31]	-*	47.6% on ATR	36.2	45.7	26 (10.2)
Meya et al. [32]	79	All naïve	No data	45.7	50 (8.2)
Rugemalila et al. [33]	96	44% on ART	No data	66	7 (3)
Longley et al. [34]	55.5	All naïve	No data	No data	28 (4.3)
Hailu et al. [35]	-**	52% on ART	45	33	9 (3.4)
Letang et al. [36]	71	All naïve	No data	No data	28 (3.7)
Christopher et al. [37]	-***	All on ART	No data	No data	33 (9.9)
Williams et al. [38]	25	51% on ART	No data	No data	149 (72)^^^
Alemu et al. [39]	123	74% on ART	100	28	31 (8.4)
Derbie et al. [40]	51.8	All on ART	No data	No data	16 (11.7)
Mamuye et al. [41]	93	51% on ART	36%	39	18 (9.1)
Oyella et al. [42]	23	All naïve	No data	37.1	69 (19)
Ogouyemi et al. [18]	-**	All naïve	No data	No data	6 (1.7)
Drain et al. [43]	75	All naïve	No data	No data	39 (9)
Mdodo et al. [44]	72	30.6% on ART	No data	80.6	111 (33)
Micol et al. [45]	24	All naïve	28%	52.5	59 (18)
Jarvis et al. [46]	97	All naïve	No data	No data	46 (7)
Wajanga et al. [47]	68	All naïve	17.9%	No data	17 (5.1)
Magambo et al. [48]	97	All naïve	66	10	10 (7.1)

Four studies didn’t report the exact median CD4 count of their study participants. However, *about 59 (23.2%) of this particular study participants had CD4 < 100; **All the study participants of this study had CD4 count < 100; *** About 121(36.3%) of this study participants had CD4 < 200

^^^**Outlier**: subjects were HIV patients suspected for meningitis who were admitted to a hospital. The figure is excluded from the pooled prevalence analysis

**Fig. 2:**
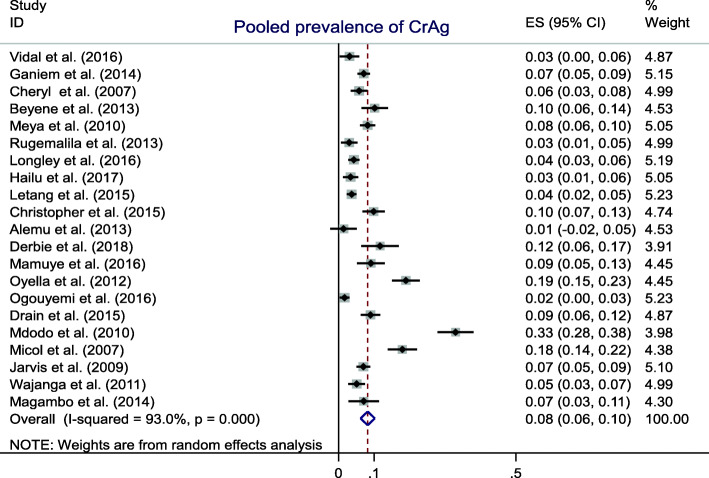
The pooled prevalence of cryptococcal antigenemia in resource limited settings, 2007–2018

The overall reported prevalence of cryptococcal antigenemia was between 1.7 and 33%. Running meta-analysis, the pooled prevalence was at 8% (95%CI: 6–10%) (Table 2 and Fig. 2). Our sub-group analysis also showed that the prevalence of cryptococcal antigenemia in Ethiopia varied between 3.4 and 11.7%. The pooled prevalence was at 7% (95%CI: 3–11%) among HIV infected patients at different ART status and CD4 count.

### Predictors of cryptococcal antigenemia

The statistically significant predictors of positive cryptococcal antigen test are depicted below in (Table 3). Body mass index< 18.5 kg/m2, CD4 count < 100 cells and male gender were reported by two or more articles as an important predictors of cryptococcal antigenemia.

**Table 3:** Reported factors associated with cryptococcal antigenemia among HIV infected patients in resource limited settings, 2007–2018

*Author (s)*	*Reported predictors for positive CrAg test*
Vidal et al. [28]	No data
Ganiem et al. [29]	No data
Cheryl et al. [30]	No data
Beyene et al. [31]	Being ART naive and ART-defaulter
Meya et al. [32]	A cryptococcal diagnosis during follow-up
Rugemalila et al. [33]	No data
Longley et al. [34]	No data
Hailu et al. [35]	Being male, living in rural areas, being hospitalized
Letang et al. [36]	No data
Christopher et al. [37]	Female gender, CD4 count of < 200 cell/μL
Williams et al. [38]	No data
Alemu et al. [39]	An increasing age, self-reported fever, CD4 count < 100 cells and site of screening.
Derbie et al. [40]	Gender
Mamuye et al. [41]	Lower median CD4, history of cryptococcal disease, having symptoms of headache, head stiffness
Oyella et al. [42]	Low body mass index, CD4+ count of less than 50 cells/mm3, recent diagnosis of HIV infection and meningeal signs
Ogouyemi et al. [18]	Body mass index< 18.5 kg/m2, an alteration of the general condition with a CD4 lymphocyte counts< 50cells/μL
Drain et al. [43]	CD4 counts < 50 cells/μL
Mdodo et al. [44]	male sex, headache, blurred vision and previous antifungal drug use
Micol et al. [45]	Countryside residence, headache, body mass index < 15.4 kg/m2, CD4+ count < 50 cells/mm3, male gender
Jarvis et al. [46]	Baseline CD4 cell count, incident cryptococcal meningitis, history of cryptococcal disease
Wajanga et al. [47]	CD4 counts of < 100 cells, altered mental status, neck stiffness, fever
Magambo et al. [48]	Age, body mass index, CD4 count and WHO stage

## Discussion

Cryptococcal meningitis, a deadly opportunistic fungal infection, is a leading cause of death among people with advanced HIV in resource limited settings [15, 49, 50]. However, it is one of the neglected topics by public health authorities while most deaths from the diseases are avoidable [23, 50–53]. There is quite few data regarding the prevalence of cryptococcosis in resource limited settings for public health measures. Therefore, in this systematic review data of some 8338 HIV positive individuals is described to uncover the prevalence of cryptococcal antigenemia and its possible predictors. The median age of the patients in the included studies range 30–40 years which implies that HIV infection continue affecting the productive segment of the population.

In this review the reported median CD4 count of the HIV patients was between 20 and 123cell/μl. Except a study [39], the rest reported mean CD4 count of the participants below 100 cells (range 23–97 cells/μl). On top of this twelve studies [18, 29, 30, 32, 34, 36, 42, 43, 45–48] reported that all the participants were ART naïve. Consequently, low CD4 count coupled with not starting ART would expose HIV infected patients for higher risk of different opportunistic infections including, cryptococcosis.

Our review result showed the overall prevalence of cryptococcal antigenemia between 1.7 and 33%; the pooled prevalence was at 8% (95%CI: 6–10%). A review by Firacative et al. (2018) on the status of cryptococcosis in Latin America reported prevalence of 10–21% [22] in the context of HIV patients with CM. Although the papers included in the present review recruited patients with different clinical ground, including their different CM status, our finding is still in line with the above report. Similarly, based on Rajasingham et al. (2017) review report on the Global burden of HIV-associated cryptococcal meningitis, the estimated global cryptococcal antigenaemia was at 6% (95%CI 5.8–6.2%) among people with a CD4 cell count of less than 100 cells per μL in 2014. This finding is in line with our pooled estimate at (8%). In addition, according to this report, the Sub-Saharan Africa accounted for 73% of the estimated cryptococcal meningitis cases in 2014. Moreover, the report also highlighted that there might be an ongoing burden of HIV-associated cryptococcal disease, primarily in sub-Saharan Africa [54]. Ford and his colleague (2018) on their review article reported the global pooled prevalence of cryptococcal antigenemia at 6.5% among HIV patients with CD4 count ≤100 cells/μL which is still comparable with our report [55].

Another review by Park et al. (2009) aimed at estimating the current global burden of cryptococcal meningitis among persons living with HIV showed an incidence ranged from 0.04 to 12% per year. Sub-Saharan Africa had the highest yearly burden estimate (median incidence at 3.2%). In contrast, the median incidence was lowest in Western and Central Europe and Oceania (</=0.1% each) [5]. This implies that the overall prevalence of the disease would be much higher, may be close to or greater than our pooled report at (8%), in poor settings. In contrast, the prevalence of cryptococcosis in the developed world has decreased as there is quite low burden of HIV and is also being diagnosed earlier, but is still significant, and the problem in resource-limited settings is exceedingly high [56] in which over half of patients die within 10 weeks of diagnosis compared to as few as 10% of patients from developed nations [57].

The pooled prevalence of cryptococcal antigenemia in Ethiopia was at 7% (95%CI: 3–11%) among HIV infected patients at different clinical context. Comparable reports have been released in Ethiopia and overseas; Bite et al. (2016) had reported (8.5%) positive cryptococcal antigenemia proportion in Addis Ababa, Ethiopia [58]. Thomsen et al. (2018) reported that, of HIV patients included in a study in Guinea-Bissau, (10%) had a positive cryptococcal antigen test [59]. On top of this, in our review the pooled prevalence of cryptococcal antigenemia was 11% (Uganda), 4% (Tanzania) and 7% (South Africa). As the HIV patients had different CD4 count, ART status and other background variables, i.e. due to clinical variability, minor variation in the prevalence of cryptococcal antigenemia is likely to happen in different settings. Further, as most of the participants had CD4 count less than 100 cells/μl, relatively higher proportion of Cryptococcal antigenemia is more likely to be reported among these immunosuppressed HIV patients in these settings [42]. In contrast to our result, a relatively lower prevalence (2.9%) of Cryptococcal antigenemia among HIV/AIDS patients was reported in United States in 2012 [21]. Difference in ART adherence and HIV care might contribute for the lower prevalence of the case in the US than most African countries.

With regard to the possible predictors of cryptococcal antigenemia, body mass index< 18.5 kg/m2, CD4 count < 100 cells, presented with headache and male gender were reported by two or more articles as an important predictors of cryptococcal antigenemia that could potentially be utilized for public health measures. These all might directly or indirectly contributed for reduced immune status of individuals that could put them at risk for different opportunistic infections, including cryptococcosis. Specifically, lower CD4 count has strong correlation with sever immune depletion, hence risk of opportunistic infections. Liechty et al. (2007) reported that among HIV-infected individuals with CD4 cell count < 100 cells/μl, cryptococcal antigenemia was associated with a higher risk of death than CrAg negative participants [30]. Other studies in different settings also reported that lower CD4 count (usually < 100 cells/mm), low body mass index, having neck pain and signs of meningeal irritation were an important predictors of cryptococcal antigenemia [18–21, 55]. Thomsen et al. (2018) reported that self-reported headache and fever were also predictors of a positive CrAg test [59].

In our review almost half of the papers reported 10–80.6% proportion of headache and 17.9 and 100% proportion of clinical stage IV HIV disease among HIV patients. Few of other papers reported these variables as an important predictor of cryptococcal antigenemia [44, 45, 47, 48]. Therefore, stakeholders and policy makers should consider target screening and management of HIV patients coming-up with such kind of associated factors to decrease the morbidity and mortality associated with cryptococcal infection in resource limited settings.

### Strength and limitations

This systematic review presented the prevalence of cryptococcal antigenemia and its predictors among HIV patients in resource limited settings, which is an added knowledge on the existing literature [55]. It includes studies from different settings: Africa, Asia and Latin America that allowed incorporating a better representation of data for policy making.

However, our review should be interpreted in light of a couple of drawbacks including but not limited to the small number of included studies despite the setting covers large number of countries. Especially, Latin America is underrepresented in this review. As a result, the absence of data from some countries might compromise the overall picture of the prevalence of cryptococcal antigenemia and its predictors for clear understanding of the problem for further considerations. The review didn’t provide data on the CrAg titer and specific cryptococcal species involved in positive CrAg tests. The other possible pitfall of this review is the variation in demographic characteristic of the study subjects (clinical variability). Finally, the other limit relates to the point in time analysis of studies with cross-sectional study designs, as majority of the articles were this type, which inherently could affect the overall picture of the magnitude of cryptococcal antigenemia. Restricting our inclusion criteria to include only articles published in English languages may have missed relevant studies and reduced the precision of our results.

## Conclusions

The pooled prevalence of cryptococcal antigenemia among HIV infected patients at different CD4 count and ART status was at 8%. Body mass index< 18.5 kg/m^2^, CD4 count < 100 cells, presenting with headache and male gender were reported by two or more articles as an important predictors of cryptococcal antigenemia. Therefore, it will be good to consider routine screening for CrAg among HIV infected patients specifically those presenting with these predictors. Policy makers should consider the implementation of targeted screening and treatment interventions for asymptomatic cryptococcal antigenemia patients in resource limited settings. Meningitis associated with cryptococcosis might be a reflection of HIV treatment programme failure; therefore timely HIV testing and rapid linkage to care will have paramount importance for patients. Finally, further work is needed to better define the scope of the problem and track the epidemiology of this infection, in order to prioritize prevention, diagnosis, and treatment strategies.

## Supplementary information

**Additional file 1 Supplement 1:** Search Strategy

## Data Availability

All the generated data in this review are included in the manuscript.

## Abbreviations

AIDS: Acquired Immunodeficiency Syndrome
ART: Anti-Retroviral Therapy (or Treatment)
*C. neoformans*: *Cryptococcus neoformans*
CD: Cluster of Differentiation
CI: Confidence Interval
CM: Cryptococcal Meningitis
CNS: Central Nervous System
CrAg: Cryptococcal Antigen
CSF: Cerebrospinal Fluid
EIA: Enzyme Immunoassay
HIV: Human Immunodeficiency Virus
JBI: Joanna Briggs Institute
LFA: Lateral Flow Assay
UNAIDS: United Nations Programme on HIV/AIDS
WHO: World Health Organization
