# Supplementary material for: Cryptococcal antigenemia and its predictors among HIV infected patients in resource limited settings: a systematic review
Source: BMC Infect Dis. 2020 Jun 11;20:407. doi: 10.1186/s12879-020-05129-w (PMC7291525; doi:10.1186/s12879-020-05129-w)
Supplement: Supplementary file 1 — Additional file 1 Supplement 1: Search Strategy [file 12879_2020_5129_MOESM1_ESM.docx]

**Supplement 1: Search Strategy**

| PubMed | Scopus |
| --- | --- |
| (((((("Meningitis, Cryptococcal"[Mesh])) OR (“Cryptococcosis"[Mesh] OR "*Cryptococcus* gattii"[Mesh] OR “*Cryptococcus* *neoformans*"[Mesh] )) AND "Risk Factors"[Mesh]) AND "HIV"[Mesh]) OR "Acquired Immunodeficiency Syndrome"[Mesh]) AND "Developing Countries"[Mesh] | ( TITLE-ABS-KEY ( meningitis )  OR  TITLE-ABS-KEY ( cryptococcal )  OR  TITLE-ABS-KEY ( cryptococcosis )  OR  TITLE-ABS-KEY ( "*Cryptococcus* gattii" )  OR  TITLE-ABS-KEY ( "cryprococcus neoformans" )  AND  TITLE-ABS-KEY ( "risk factor" )  AND  TITLE-ABS-KEY ( hiv )  OR  TITLE-ABS-KEY ( "Acquired immnune deficiency syndrome" )  AND  TITLE-ABS-KEY ( "developing countries" ) )  AND  ( LIMIT-TO ( DOCTYPE ,  "ar" ) )  AND  ( LIMIT-TO ( LANGUAGE ,  "English" ) )  AND  ( LIMIT-TO ( SRCTYPE ,  "j" ) ) |
